# Gender- and age-specific associations of childhood maltreatment with peripheral serum inflammatory cytokines in middle school students

**DOI:** 10.3389/fimmu.2023.1067291

**Published:** 2023-01-31

**Authors:** Zhengge Jin, Shuqin Li, Ruoyu Li, Xianbing Song, Shichen Zhang, Ying Sun, Fangbiao Tao, Yuhui Wan

**Affiliations:** ^1^ Department of Maternal, Child & Adolescent Health, School of Public Health, Anhui Medical University, Hefei, Anhui, China; ^2^ Anhui Provincial Key Laboratory of Population Health & Aristogenics, Hefei, Anhui, China; ^3^ Department of Human Anatomy, Histology & Embryology, Anhui Medical College, Hefei, Anhui, China

**Keywords:** childhood maltreatment, inflammatory cytokines, gender, age, middle school students

## Abstract

**Background:**

The impact of childhood maltreatment on multiple inflammatory cytokines among middle school students remains to be elucidated. This study aimed to examine the associations of different types of childhood maltreatment with peripheral serum inflammatory cytokines (interleukin-10, interleukin-1β, interleukin-6, interleukin-8, and tumor necrosis factor-α) in middle school students, and to explore the differences in these associations between boys and girls and between late (≥15 and<20 years) and early (≥11 and <15 years) adolescence.

**Methods:**

A total of 1122 students were recruited from a boarding middle school. Each participant was asked to respond to a detailed questionnaire on childhood maltreatment, from whom one blood sample was drawn *via* venous blood.

**Results:**

In the overall sample there was no association between childhood maltreatment and peripheral serum inflammatory cytokines; (2) emotional abuse was significantly correlated with IL-1β only in girls (*B* = -0.16; 95% CI, -0.28~-0.03; *p* = 0.06); (3) in late adolescence, emotional abuse, emotional neglect, and childhood maltreatment had marked link with IL-8 (*B* = 0.39; 95%CI, 0.16~0.63; *p* = 0.01; *B* =0.20; 95% CI, 0.04~0.37; *p* = 0.08; *B* = 0.50; 95% CI, 0.18~0.82; *p* = 0.01, respectively).

**Conclusion:**

These findings also strengthened an inference regarding the effects of childhood maltreatment on inflammation of students in late adolescence.

## Introduction

Inflammation, the response of body tissues to injury, is protective against various bacterial and viral infections (1). However, low-grade, chronic inflammation can persist for a long time, contributing to the pathogenesis of other human diseases. For example, inflammatory processes have also been prospectively linked to the pathogenesis of depression (2–4). Moreover, evidence suggests that chronic inflammation is a significant risk predictor of cardiovascular diseases (5) as well as a biological marker for development and progression of cancer (6). In addition, chronic inflammation is the leading underlying cause of death (7, 8). Some studies have indicated that the elevated levels of inflammation are linked to experiences of childhood adversities, stress, and trauma (9–11).

Childhood maltreatment has been shown to result in a host of harmful outcomes immediately and throughout the lifespan (12–14), and systemic inflammation is considered a potential biological mechanism mediating this association (15). Based on the situation, some cytokines are involved in both pro-inflammatory and anti-inflammatory activities. Cytokines that promote the inflammatory cascade are called pro-inflammatory mediators, including interleukin (IL)-6, IL-8, IL-1β, tumor necrosis factor-α (TNF-α), *etc.* The production of anti-inflammatory cytokines such as IL-4, IL-10, IL-11, and IL-13 blocks this process by regulating the pro-inflammatory cytokine response (16). In recent years, relevant studies have shown that IL-1β, IL-6, IL-8, TNF-α and IL-10, as parameters reflecting the level of inflammation in the body, are good indicators of immune dysfunction and changes in the level of inflammatory factors (10, 17–19). Many studies have elucidated the relationship between a single type of childhood maltreatment and inflammation, and it is likely that different types of childhood maltreatment manifest different associations with inflammation. Based on previous research, adults with a documented history of childhood maltreatment, especially physical abuse, were prone to increased risk of higher levels of inflammatory cytokines (C-reactive protein [CRP]) than non-maltreated individuals were (10). Some systematic reviews and meta-analyses also demonstrated that adults exposed to childhood trauma (sexual, physical, or emotional abuse) may have apparently elevated baseline peripheral levels of CRP, IL-6, and TNF-α (17, 20). Childhood trauma (emotional abuse, physical abuse, sexual abuse and emotional neglect before the age of 16) was associated with increased levels of lipopolysaccharide-stimulated cytokines, such as IL-10, IL-6, and IL-8, with evidence for a dose-response relationship (21). According to a study with the application of longitudinal data from the Multidimensional Assessment of Preschoolers Study, substance abuse was correlated to higher levels of pro-inflammatory markers such as IL-1β and IL-6 (18). Focusing on the single adversity of childhood maltreatment may help to sort out the life-course mechanisms involved (19), yet the impact of such adversity may be confounded by the experience of other adversities excluded in the analysis. Therefore, the associations of different types of childhood maltreatment with inflammatory markers call for further exploration.

Furthermore, the potential gender difference remains an interesting focus in the link between childhood maltreatment and inflammatory biomarkers. As indicated in some research, women have greater inflammatory responses to childhood stressors (22, 23), while others document no gender difference in the effect of childhood maltreatment on inflammatory dysregulation (17). Apparently, whether the impacts vary by gender remains unclear. On the other hand, for a better implementation of interventions in schools, it is necessary to determine whether gender difference exists in the relationship between childhood maltreatment and inflammatory cytokines among middle school students under the influence of school settings. Links between child maltreatment and low-grade inflammation in adulthood seemed to be well documented (24, 25); however, only a handful of studies dwelt on the correlation between child maltreatment and low-grade inflammation in adolescents (11, 26). As the immune system advances rapidly in adolescence, it is crucial to characterize the progression of the inflammatory process in adolescents concerning child maltreatment.

Despite the clarification of the relationship between childhood abuse and inflammatory factor levels in former studies (10, 15–21), similar evidence is still lacking in China. Worse still, inconsistency could be observed in the previous evidence found in the association affected by different gender and age groups (11, 17, 22–26). To address this gap in the roles of gender and age in the relationship between childhood maltreatment and inflammation, we made a preliminary investigation on the relationship between childhood maltreatment and inflammatory profiles [i.e., pro-inflammatory (IL-1β, IL-6, IL-8, TNF-α) and anti-inflammatory (IL-10)] in middle school students, with an examination of the discrepancy in the patterns between boys and girls and between students in early and late adolescence.

## Materials and methods

### Sample and procedures

From October to December, 2018, a survey was conducted on a sample of 1312 students selected from a boarding school in Shenyang City, Liaoning Province, China, where all juniors and seniors were recruited. An anonymous questionnaire was required to be completed, and a blood sample of each participant was provided through a venous blood draw in cooperation with doctors. Besides, the survey was conducted in classrooms by investigators during extracurricular hours. Teachers, though excluded from the survey, were invited to be in charge of maintaining classroom discipline. The objective of the survey was introduced, the questionnaires offered, and the principles of anonymity and confidentiality of participation were emphasized. Investigators were on the spot in case questions from students arose during the survey. The ID number of students was used for the only identification of their questionnaires and blood samples. The sober venous blood draw was performed within two to three days before and after the completion of the questionnaires.

Due to an unwillingness to respond to the questionnaire, absence from school, high levels of missing data (questionnaires with missing values >5% were eliminated), or obviously fictitious responses, some students failed to contribute, and eventually, 1203 questionnaires were recovered out of 1312 students. A total of 1182 blood samples were obtained because of the following reasons (1): some students had anemia, cough, or fever (n = 7); (2) some students who were interviewed declined to have blood drawn (n = 115); and (3) when the inflammatory factor level of a sample exceeded the mean value ±3 times the standard deviation range, the inflammatory factor level was treated as a missing value (n = 8).

The study design and data collection procedures were approved by the Ethics Committee of Anhui Medical University (20170290). Informed consent was obtained from both the students and their parents or guardians.

### Measures

#### Childhood maltreatment

Childhood maltreatment was evaluated *via* using the Childhood Trauma Questionnaire (CTQ) (27), a widely used 28-item tool to assess five forms of childhood trauma (physical abuse, sexual abuse, emotional abuse, physical neglect, and emotional neglect). The CTQ was translated and validated in Chinese (28). The participants were interviewed about their abusive childhood experiences before entering middle school. Response scores ranged from 1 to 5 (never true, rarely true, sometimes true, often true, and very often true, respectively). The higher the scores, the more severe childhood maltreatment one received. Cronbach’s α coefficient of the emotional abuse, physical abuse, sexual abuse, emotional neglect, physical neglect, and childhood maltreatment scale in this study was 0.692, 0.731, 0.767, 0.804, 0.637, and 0.768, respectively.

#### Inflammation

Pro-inflammatory cytokines (IL-1β, IL-6, IL-8, and TNF-α) and anti-inflammatory cytokines (IL-10) were collected from the fasting serum from 7:00 to 9:00 in the morning. The detailed procedure was conducted as follows. The venous blood samples of 1182 participants were placed into tubes. The blood was allowed to clot for at least 30 minutes before centrifugation for 10 minutes at 1000 × g. The serum was immediately removed, and the samples were stored at ≤-20°C. Serum cytokine levels were measured using the Human High Sensitivity T Cell Magnetic Bead Panel Protocol from the Milliplex^®^ MapKit (cat. no. HSTCMAG-28SK, USA), following the manufacturer’s instructions. Briefly, assay plates were washed with wash buffer, sealed and mixed on a plate shaker for 10 min at room temperature. The wash buffer was decanted, and 50 μL of the diluted standards, quality controls, and serum samples were added to the appropriate wells. After the addition of samples or controls, the plates were incubated overnight at 4°C on a plate shaker with fluorescently labeled capture antibody-coated beads. After overnight incubation with capture antibodies to detect IL-1β, IL-6, IL-8, TNF-α, and IL-10, the well content was removed and washed using a handheld magnet. Followed by the addition of 150 μL drive fluid to all wells, the sample was run on MAGPIX^®^ with xPONENT^®^ software (Luminex Corporation, Austin, Texas, USA). Analysis of the cytokine median fluorescence intensity was performed using Milliplex Analyst version 5.1. The intra- and inter-assay coefficients of variation for all tested cytokines were <5% and <15-20%, respectively.

#### Covariates

Based on the previous investigation of the research group (29, 30), the covariates in the multivariate analyses were sociodemographic characteristics, including age, gender, urban/rural residence, parental education level (middle school and below, high school and above), and perceived economic status of the family (poor, moderate, or good). Body mass index was also adjusted, which was calculated according to objectively measured height and weight.

#### Statistical analyses

Sociodemographic data and childhood maltreatment were described in the total population. Five forms of childhood abuse and serum cytokine values (TNF-α, IL-6, IL-1β) were natural log transformed to better approximate the normality of the residuals. Measurement data were expressed as mean ± standard deviation (mean ± SD), and two independent samples t-tests were used to compare the log-transformed inflammatory cytokine levels in different genders and ages. Linear regression analyses were performed to examine the association between childhood maltreatment and inflammatory cytokine levels. We also examined the associations of specific maltreatment subtypes (emotional abuse, physical abuse, sexual abuse, emotional neglect, and physical neglect). Given the literature on gender and age differences, separate regression analyses were conducted, with the sample split first by gender (boys *vs*. girls) and then by age [late adolescence (≥15 and<20 years) *vs*. early adolescence (≥11 and <15 years)] (31). Moderation analyses were used to assess the moderating role of gender and age in the relationships between child maltreatment and inflammatory markers. All analyses were conducted using SPSS version 23.0. The threshold for statistical significance was set at *p*< 0.05. To reduce the false-positive rate, we used the false discovery rate (FDR) approach with a cut-off of 0.10.

## Results

### Descriptive characteristics of samples

**Table 1** showed the descriptive data on the distribution of age, gender, urban/rural residence, parental education level, perceived economic status of the family, BMI, and log-transformed child maltreatment.

**Table 1:** Sample characteristics and descriptive statistics.

Variables	Mean ± SD or n (%)
**Age (years)**	15.38 ± 1.85
Gender	
Boys	628 (55.97)
Girls	494 (44.03)
Urban/rural residence	
Urban	508 (45.28)
Rural	614 (54.72)
Father’s education level	
Middle school and below	585 (52.14)
High school and above	537 (47.86)
Mother’s education level	
Middle school and below	585 (52.14)
High school and above	537 (47.86)
Perceived economic status of the family	
Poor	97 (8.65)
Moderate	769 (68.54)
Good	256 (22.82)
**BMI (kg/m^2^)**	22.41 ± 4.70
Child maltreatment	
In (Emotional abuse)	0.81 ± 0.14
In (Physical abuse)	0.74 ± 0.10
In (Sexual abuse)	0.71 ± 0.07
In (Emotional neglect)	0.86 ± 0.19
In (Physical neglect)	0.83 ± 0.15
In (Child Maltreatment)	1.51 ± 0.10

### The differences in peripheral serum inflammatory cytokine levels by gender and age

**Table 2** showed that the serum levels of IL-10 and IL-1β in boys were higher than in girls on average, and serum IL-8 and TNF-α in girls were higher than those in boys with statistical differences (*p*<0.05). Meanwhile, the serum levels of IL-1β, IL-6 and TNF-α in students in late adolescence were higher than those in early adolescence on average, with statistical differences (*p*<0.05). The statistical differences were enhanced after FDR adjustment (adjusted *p*<0.001, **Supplementary Table A1**). Further analysis of the log-transformed inflammatory cytokine distributions by gender and age was presented in **Supplementary Figure 1**.

**Table 2:** Inflammatory cytokine characteristics by gender and age.

Inflammatory cytokines	Gender		t-value	P-value	Age		t-value	P-value
	Boys (n=628)	Girls (n=494)			≥11 and <15 years (n=459)	≥15 and<20 years (n=663)		
In (IL-10)	1.18 ± 0.17	1.22 ± 0.16	-3.851	**<0.001**	17.22 ± 6.81	16.65 ± 6.42	1.567	0.117
In (IL-1β)	-0.05 ± 0.23	0.00 ± 0.20	-4.649	**<0.001**	0.94 ± 0.40	1.11 ± 0.47	-5.148	**<0.001**
In (IL-6)	0.55 ± 0.33	0.56 ± 0.28	-0.785	0.433	4.32 ± 4.55	5.13 ± 5.37	-2.945	**0.004**
In (IL-8)	1.28 ± 0.39	1.22 ± 0.40	2.595	**0.010**	24.00 ± 19.79	26.42 ± 21.80	-1.253	0.210
In (TNF-α)	0.69 ± 0.18	0.62 ± 0.20	6.618	**<0.001**	4.69 ± 1.94	5.21 ± 2.25	-3.463	**0.001**

All statistically significant values were bolded.

### Associations of childhood maltreatment with the levels of peripheral serum inflammatory cytokines in the total sample

**Table 3** showed that in the overall sample, despite the introduction of all control variables, the association between emotional abuse with elevated levels of IL-6 (*B* = 0.15, 95% CI, 0.01~0.28) disappeared after adjustment for FDR (**Table A2**).

**Table 3:** Associations of childhood maltreatment with the levels of peripheral serum inflammatory cytokines in the total sample.

Childhood maltreatment	IL-10					IL-1β					IL-6					IL-8					TNF-α				
	*B*	95%*CI*	*SE*	*t*	*P*	*B*	95%*CI*	*SE*	*t*	*P*	*B*	95%*CI*	*SE*	*t*	*P*	*B*	95%*CI*	*SE*	*t*	*P*	*B*	95%*CI*	*SE*	*t*	*P*
Emotional abuse	-0.01	-0.08~0.06	0.04	-0.23	0.82	-0.07	-0.16~0.02	0.05	-1.56	0.12	**0.15**	**0.01**~**0.28**	**0.07**	**2.13**	**0.03**	0.10	-0.08~0.27	0.09	1.10	0.27	-0.02	-0.10~0.07	0.04	-0.38	0.70
Physical abuse	-0.06	-0.16~0.04	0.05	-1.18	0.24	-0.09	-0.22~0.03	0.07	-1.44	0.15	0.09	-0.10~0.28	0.10	0.93	0.35	0.03	-0.21~0.27	0.12	0.23	0.82	-0.08	-0.19~0.32	0.06	-1.41	0.16
Sexual abuse	-0.04	-0.18~0.11	0.07	-0.48	0.63	-0.03	-0.22~0.16	0.10	-0.31	0.76	0.07	-0.22~0.35	0.15	0.45	0.65	0.04	-0.31~0.38	0.18	0.21	0.84	-0.01	-0.18~0.15	0.08	-0.15	0.88
Emotional neglect	0.03	-0.03~0.08	0.03	0.98	0.33	0.04	-0.03~0.11	0.03	1.15	0.25	0.03	-0.07~0.13	0.05	0.64	0.52	0.09	-0.04~0.21	0.06	1.36	0.18	0.01	-0.05~0.07	0.03	0.27	0.79

All statistically significant values were bolded.

### Gender-specific associations of childhood maltreatment with the levels of peripheral serum inflammatory cytokines

In separate gender-specific regressions (**Table 4**), which were adjusted for age, urban/rural, parental education level, perceived family economic status, and BMI, girls with increased emotional abuse scores showed decreased IL-1β levels (*B* = -0.16; 95% CI, -0.28~-0.03), but the relationship was attenuated after FDR was adjusted (adjusted *P*=0.06, **Supplementary Table A3**). In addition, boys with emotional abuse scores showed elevated inflammatory IL-6 (*P*=0.05), but the relationship was attenuated after FDR was adjusted (adjusted *P*=0.28, **Supplementary Table A3**). The findings indicated that no moderated roles were detected in the relationships between types of child maltreatment and inflammatory cytokine levels by gender.

**Table 4:** Gender-specific associations of childhood maltreatment with the levels of peripheral serum inflammatory cytokines.

Childhood maltreatment	IL-10					IL-1β					IL-6					IL-8					TNF-α				
	*B*	95%*CI*	*SE*	*t*	*P*	*B*	95%*CI*	*SE*	*t*	*P*	*B*	95%*CI*	*SE*	*t*	*P*	*B*	95%*CI*	*SE*	*t*	*P*	*B*	95%*CI*	*SE*	*t*	*P*
Boys																									
Emotional abuse	0.02	-0.08~0.12	0.05	0.37	0.71	-0.01	-0.14~0.12	0.07	-0.09	0.93	**0.20**	**0.00**~**0.39**	**0.10**	**2.00**	**0.05**	0.08	-0.15~0.32	0.12	0.71	0.48	-0.07	**-**0.17~0.04	0.05	-1.19	0.24
Physical abuse	-0.06	-0.18~0.06	0.06	-0.96	0.34	-0.09	-0.25~0.07	0.08	-1.11	0.27	0.04	-0.20~0.28	0.12	0.32	0.75	-0.09	-0.37~0.19	0.14	-0.63	0.53	-0.08	-0.21~0.05	0.07	-1.18	0.24
Sexual abuse	-0.04	-0.19~0.12	0.08	-0.45	0.65	-0.03	-0.24~0.18	0.11	-0.29	0.78	0.07	-0.25~0.40	0.17	0.45	0.66	-0.02	-0.39~0.35	0.19	-0.09	0.93	-0.04	-0.22~0.13	0.09	-0.50	0.62
Emotional neglect	-0.01	-0.08~0.06	0.04	-0.16	0.87	0.05	-0.05~0.14	0.05	0.97	0.34	0.00	-0.14~0.14	0.07	0.03	0.98	0.07	-0.09~0.24	0.08	0.87	0.39	-0.00	-0.08~0.07	0.04	-0.08	0.94
Physical neglect	-0.08	-0.17~0.01	0.04	-1.80	0.07	0.03	-0.08~0.15	0.06	0.57	0.57	-0.01	-0.19~0.16	0.09	-0.15	0.88	0.02	-0.19~0.23	0.11	0.20	0.84	0.00	-0.09~0.10	0.05	0.03	0.98
Childhood maltreatment	-0.06	-0.19~0.07	0.06	-0.93	0.35	0.01	-0.16~0.18	0.09	0.14	0.89	0.04	-0.21~0.29	0.13	0.34	0.73	0.09	-0.21~0.38	0.15	0.57	0.57	-0.04	-0.18~0.09	0.07	-0.62	0.54
Girls																									
Emotional abuse	-0.03	-0.13~0.07	0.05	-0.57	0.57	**-0.16**	**-0.28**~**-0.03**	**0.06**	**-2.48**	**0.01**	0.08	-0.10~0.26	0.09	0.84	0.40	0.08	-0.17~0.34	0.13	0.63	0.53	0.04	-0.09~0.17	0.06	0.63	0.53
Physical abuse	-0.06	-0.24~0.12	0.09	-0.64	0.52	-0.20	-0.43~0.03	0.12	-1.69	0.09	0.18	-0.16~0.52	0.17	1.03	0.31	0.29	-0.19~0.76	0.24	1.20	0.23	-0.11	-0.34~0.12	0.12	-0.94	0.35
Sexual abuse	0.00	-0.40~0.41	0.21	0.01	0.99	-0.11	-0.63~0.40	0.26	-0.44	0.66	-0.02	-0.80~0.77	0.40	-0.04	0.97	0.33	-0.71~1.37	0.53	0.62	0.53	0.19	-0.32~0.70	0.26	0.74	0.46
Emotional neglect	0.07	-0.01~0.14	0.04	1.78	0.08	0.04	-0.06~0.13	0.05	0.80	0.43	0.08	-0.06~0.22	0.07	1.16	0.25	0.11	-0.08~0.30	0.10	1.17	0.24	0.02	-0.07~0.12	0.05	0.49	0.62
Physical neglect	0.05	-0.05~0.15	0.05	0.93	0.35	0.00	-0.12~0.13	0.07	0.05	0.96	0.07	-0.11~0.26	0.09	0.77	0.44	-0.03	-0.29~0.23	0.13	-0.22	0.83	-0.04	-0.17~0.08	0.07	-0.67	0.51
Childhood maltreatment	0.07	-0.07~0.21	0.07	0.94	0.35	-0.05	-0.23~0.13	0.09	-0.59	0.56	0.16	-0.10~0.43	0.13	1.21	0.23	0.17	-0.20~0.54	0.19	0.92	0.36	0.01	-0.18~0.19	0.09	0.05	0.96

All statistically significant values were bolded.

### Age-specific associations of childhood maltreatment with the levels of peripheral serum inflammatory cytokines

In separate regressions for late and early adolescence (**Table 5**), which were adjusted for gender, urban/rural, parental education level, perceived family economic status, and BMI, emotional abuse was associated with significantly lower odds of elevated IL-1β levels among students in early adolescence (*B* = -0.14; 95% CI, -0.26~-0.01). However, for those in late adolescence, emotional abuse was positively associated with elevated IL-6 (*B* = 0.19; 95% CI, 0.00~0.38) and IL-8 (*B* = 0.39; 95%CI, 0.16~0.63), physical abuse was positively associated with increased IL-6 levels (*B* = 0.37; 95% CI, 0.04~0.69), emotional neglect was positively associated with uplifted IL-8 levels (*B* = 0.20; 95% CI, 0.04~0.37), and childhood maltreatment was linked to a significantly higher risk of raised IL-8 levels (*B* = 0.50; 95% CI, 0.18~0.82). But the correlation between emotional abuse and IL-1β levels in early adolescence presented no statistical significance after FDR adjusted (adjusted *P* = 0.24). Besides, the relationship between emotional abuse and physical abuse and elevated IL-6 levels was attenuated after FDR adjusted (**Supplementary Table A4**). These findings indicated that the moderating roles of age were detected in the association between emotional abuse, emotional neglect and child maltreatment and IL-8 (*B* = 0.39, 0.20 and 0.50 respectively, *P* < 0.1 for all).

**Table 5:** Age-specific associations of childhood maltreatment with the levels of peripheral serum inflammatory cytokines.

Childhood maltreatment	IL-10					IL-1β					IL-6					IL-8					TNF-α				
	*B*	95%*CI*	*SE*	*t*	*P*	*B*	95%*CI*	*SE*	*t*	*P*	*B*	95%*CI*	*SE*	*t*	*P*	*B*	95%*CI*	*SE*	*t*	*P*	*B*	95%*CI*	*SE*	*t*	*P*
≥11 and <15 years																									
Emotional abuse	-0.01	-0.11~0.10	0.05	-0.11	0.92	**-0.14**	**-0.26**~**-0.01**	**0.07**	**-2.11**	**0.04**	0.09	-0.10~0.27	0.09	0.93	0.35	-0.18	-0.42~0.06	0.12	-1.51	0.13	-0.01	**-**0.12~0.10	0.06	-0.17	0.87
Physical abuse	-0.10	-0.22~0.03	0.06	-1.54	0.12	-0.08	-0.23~0.08	0.08	-0.97	0.34	-0.04	-0.25~0.18	0.11	-0.33	0.75	-0.09	-0.38~0.21	0.15	-0.58	0.56	-0.06	-0.19~0.08	0.07	-0.84	0.40
Sexual abuse	-0.02	-0.21~0.18	0.10	-0.17	0.86	-0.13	-0.38~0.12	0.13	-1.00	0.32	0.02	-0.34~0.37	0.18	0.09	0.93	-0.13	-0.60~0.34	0.24	-0.55	0.58	-0.08	-0.30~0.14	0.11	-0.74	0.46
Emotional neglect	0.03	-0.05~0.10	0.04	0.67	0.51	0.04	-0.06~0.14	0.05	0.80	0.43	0.02	-0.12~0.15	0.07	0.25	0.81	-0.04	-0.22~0.14	0.09	-0.43	0.67	0.06	-0.02~0.14	0.04	1.39	0.17
Physical neglect	-0.04	-0.14~0.06	0.05	-0.76	0.45	-0.00	-0.13~0.13	0.06	-0.02	0.99	0.02	-0.15~0.20	0.09	0.26	0.79	-0.12	-0.36~0.11	0.12	-1.01	0.31	-0.03	-0.13~0.08	0.06	-0.46	0.64
Childhood maltreatment	-0.03	-0.17~0.10	0.07	-0.46	0.65	-0.08	-0.25~0.10	0.09	-0.86	0.39	0.02	-0.23~0.26	0.12	0.12	0.90	-0.21	-0.53~0.11	0.16	-1.29	0.20	0.02	-0.13~0.17	0.08	0.25	0.80
≥15 and<20 years																									
Emotional abuse	0.00	-0.09~0.10	0.05	0.03	0.98	-0.03	-0.15~0.10	0.06	-0.41	0.68	**0.19**	**0.00**~**0.38**	**0.10**	**2.00**	**0.05**	**0.39**	**0.16**~**0.63**	**0.12**	**3.26**	**0.00**	-0.01	-0.12~0.11	0.06	-0.12	0.91
Physical abuse	0.01	-0.15~0.17	0.08	0.08	0.93	-0.11	-0.32~0.10	0.11	-1.02	0.31	**0.37**	**0.04**~**0.69**	**0.17**	**2.18**	**0.03**	0.35	-0.04~0.74	0.20	1.78	0.08	-0.08	-0.26~0.11	0.10	-0.78	0.43
Sexual abuse	-0.05	-0.25~0.16	0.11	-0.43	0.67	0.06	-0.21~0.33	0.14	0.43	0.67	0.13	-0.32~0.58	0.23	0.56	0.57	0.24	-0.27~0.74	0.26	0.92	0.36	0.06	-0.19~0.30	0.13	0.47	0.64
Emotional neglect	0.03	-0.04~0.10	0.04	0.83	0.41	0.04	-0.05~0.13	0.05	0.81	0.42	0.04	-0.09~0.18	0.07	0.60	0.55	**0.20**	**0.04**~**0.37**	**0.09**	**2.37**	**0.02**	-0.03	-0.11~0.06	0.04	-0.62	0.53
Physical neglect	-0.02	-0.11~0.07	0.05	-0.37	0.71	0.02	-0.09~0.14	0.06	0.38	0.70	0.01	-0.17~0.19	0.09	0.08	0.94	-0.04	-0.10~0.03	0.11	1.07	0.29	-0.01	-0.12~0.10	0.05	-0.16	0.87
Childhood maltreatment	0.02	-0.11~0.15	0.07	0.30	0.77	0.03	-0.14~0.20	0.09	0.32	0.75	0.16	-0.10~0.43	0.13	1.23	0.22	**0.50**	**0.18**~**0.82**	**0.16**	**3.06**	**0.00**	-0.05	-0.20~0.11	0.08	-0.57	0.57

All statistically significant values were bolded.

## Discussion

The present study explored the relationship between childhood maltreatment and inflammatory cytokines. Besides, stratification by gender and age highlighted the relevance of child maltreatment and inflammatory cytokines. This study mainly identified that girls with increased emotional abuse scores showed decreased IL-1β levels. Further, we identified that emotional abuse, emotional neglect, and childhood maltreatment was positively associated with increased IL-8 levels in late adolescence.

However, our study failed to respectively evaluate the associations of child maltreatment across dimensions with inflammatory cytokines in the whole sample, which proved to be conflicting with the findings of some previous literature (32, 33), possibly due to the fact that participants were generally healthy middle school students rather than clinical patients. The results might also have been influenced by notable methodological limitations, such as sample size and the timing of the childhood maltreatment, diet, sleep, and other confounding factors that were not addressed in this study, either. A recent systematic review showed that child trauma (sexual, physical, or emotional abuse) was associated with elevated levels of inflammatory markers like CRP, IL-6, and TNF-α (17). This finding further supported data from an earlier systematic review, in which strong evidence was detected only for associations between child maltreatment and CRP, though the authors noted that no similar association could be found in several studies on IL-6 (24). Also, a positive association was observed in some studies between early adversities and at least one measure of *in vitro* production of inflammatory cytokines (34, 35), many maltreated individuals avoided these outcomes, and negative associations could be found between early adversities and *in vitro* production of pro-inflammatory cytokines (36, 37). For example, research by Wright et al. demonstrated that a higher stress level in early childhood was significantly linked with increased production of TNF-α, suggesting a trend between more stress and less interferon-γ production (37). Thus, the correlation between different types of child maltreatment and diverse inflammatory cytokines deserves further exploration.

The present study also revealed a converse association between emotional abuse and lower IL-1β levels in girls, whereas no significant relation was detected in boys. IL-1β, produced by central and peripheral immune cells, is a key proinflammatory cytokine involved in local and systemic inflammation (38). The neural immune network hypothesis pointed out that early stress caused a low inflammatory state of the body by affecting HPA axis activity and sympathetic nervous system response, and IL 1β could cross the blood-brain barrier to induce inflammation of small glial cells in the brain, affecting neuronal plasticity, which in turn predisposed to suicidal behavior, major depression, post-traumatic stress disorder, panic disorder, bipolar disorder, and other psychological and behavioral problems (3, 25, 39–42). Our results contradicted previous data on the association between childhood trauma and IL-1β levels (43, 44). Childhood maltreatment may exert pro-inflammatory effects on immune cells throughout development. This might occur due to the association between increased stress during childhood and parallel increases in activity of the HPA axis and its end-product, cortisol (45). After binding to glucocorticoid receptors in healthy immune cells, cortisol inhibited the production of inflammatory cytokines (46). Therefore, measured by circulating markers, the pro-inflammatory phenotype of children may be masked by the upregulation of the HPA axis. In fact, the HPA axis was often socially adjusted by caregivers during much of childhood, so that the youth may exhibit attenuated HPA-axis activity around supportive caregivers (47). Thus, the students exposed to higher levels of childhood maltreatment may demonstrate lower levels of circulating inflammatory markers. Gender differences in the association between child maltreatment and inflammatory cytokines have been currently controversial. For instance, Osborn and Widom reported an apparent correlation between childhood trauma and inflammation among women instead of among men (10). A recent review examining the gender-specific effects of early life stress on inflammation (22) clarified that the association between childhood maltreatment and inflammation was driven by significant links in females, not in males. The result of this study supported the above evidence. Adolescence is a period of rapid growth and development culminating in full reproductive maturation. Major anabolic regulators induce marked changes in physiology and metabolism, leading to the pubertal transition and subsequent changes in body composition, a process in which many confounding factors affect the growth of adolescents. Teenagers’own hormone levels like reproductive hormones, testosterone and estrogen, and insulin may also take effect, and thereby conceivably influencing the levels of inflammatory cytokines. For example, testosterone in androgens is involved in the negative biofeedback mechanism of HPA, inhibiting the secretion of pro-gonadotropic hormones. Androgens (i.e., testosterone) have immune-modulating properties compared with estrogens, which may suppress the expression of the pro-inflammatory cytokines (48). On the contrary, a prospective study failed to detect the link between interpersonal stressors and circulating levels of CRP or IL-6 in a sample of female adolescents (mean age, 17 years) (49). Similarly, some studies could not find such notable gender differences in the effect of childhood adversity on physical health outcomes (17, 50). Consequently, whether the association between adolescent childhood maltreatment with inflammation is gender specific requires more far-reaching research.

After adjusting for other covariates, we observed positive associations between emotional abuse, emotional neglect, childhood maltreatment and IL-8 in late adolescence. As a proinflammatory cytokine, IL-8, mainly produced by macrophages and microglia (51), played a regulatory role in the immune response of the central nervous system. Studies proved that IL-8 could interfere with neurobiological processes during critical periods of brain development (52). Increased serum levels of 1L-8 led to dysfunctional cytokine signaling, which could induce apparent abnormal behavioral changes and various psychiatric diseases, including the onset of depression, anxiety, and cognitive dysfunction (3). The biological embedding model suggests that adverse experiences suffered by the organism during sensitive periods of growth and development can be “programmed” into macrophages through epigenetic markers, post-translational modifications and tissue remodeling, thus rendering these cells pro-inflammatory (53). Notably, we identified that the association between childhood maltreatment and IL-8 was only in late adolescence. A possible explanation is that the inflammatory system remains resilient during early adolescence, adapting to the environment and maintaining homeostasis in the body (54). This also may be due to unmeasured the timing of the childhood maltreatment on the results may be masked. Research by Hung et al. (55) demonstrated that inflammation accumulated with age, even after adjusted for confounding factors such as education, BMI, physical activity, alcohol use, and smoking status. Puberty may be the time when a pro-inflammatory phenotype appears in the circulating markers of immune function due to increases in risky behaviors, such as smoking, lack of exercise, unhealthy diet, and impulsive behaviors that increase stress exposure (56–58). Thus, as children developed into young adults (*e.g.*, college students), the immune system received more biological and behavioral inputs which induced the production and regulation of inflammation, leading to more reliable elevations in circulating inflammatory markers among at-risk populations (with early life adversity exposure) (26). This observation may remind future researchers of the significance of taking developmental processes into account in studies of inflammatory processes in children. In conclusion, our findings put forward a hint that some other factors like age may be more pertinent in affecting inflammatory cytokine levels.

### Strengths and limitations

Significant as the study of childhood maltreatment in relation to inflammatory markers in middle school students was, the main contribution of this study lay in the revelation of the roles of gender and age in the relationship between childhood maltreatment and inflammation. Besides, the large sample size and high response rate of participants were noteworthy. However, several limitations could not be overlooked in the interpretation of our results. First, the study design was cross-sectional, and the temporal ordering of the variables remained unclear, although our findings pertaining to the association between childhood maltreatment and inflammatory markers were similar to those of a previous cohort study (18). Second, due to the same background of participants in one boarding school, apparently, the findings might not be applied to all adolescents. Finally, because of the complexity of the influencing factors of inflammation, many confounding variables, such as the timing of the childhood maltreatment, sleep, diet, and recent stressful life events, may also affect inflammation, which was not included in this study, however. Despite these limitations, our study added to the literature on childhood maltreatment associated with an inflammatory pathway.

### Conclusions

This study reveals that girls with elevated scores of childhood maltreatment are more likely to show changes in the levels of inflammatory markers; students in late adolescence are particularly more susceptible to low-grade inflammation. These results strengthen an inference concerning the effects of childhood maltreatment on inflammation in middle school students and have implications for the ways in which childhood maltreatment can be incorporated into health programs in adolescents.

## Data availability statement

The original contributions presented in the study are included in the article/**Supplementary Material**. Further inquiries can be directed to the corresponding author.

## Ethics statement

The studies involving human participants were reviewed and approved by the Ethics Committee of Anhui Medical University (20170290). Written informed consent to participate in this study was provided by the participants’ legal guardian/next of kin.

## Author contributions

ZJ, SL, and RL contributed to data acquisition, data analysis and the writing of this manuscript. XS contributed to help with sample collection and data analysis. SZ, YS, and FT assisted in revising the manuscript. The present study was conceived and designed by YW. All authors contributed to the article and approved the submitted version.
