# Supplementary material for: Gender- and age-specific associations of childhood maltreatment with peripheral serum inflammatory cytokines in middle school students
Source: Front Immunol. 2023 Jan 31;14:1067291. doi: 10.3389/fimmu.2023.1067291 (PMC9927207; doi:10.3389/fimmu.2023.1067291)
Supplement: Supplementary file 1 [file DataSheet_1.pdf]

Table A1 Inflammatory cytokines characteristics by gender and age

Inflammatory Cytokines	Gender		t-value	*P*-value*	Age		t-value	*P*-value*
	Boys(n=628)	Girls(n=494)			≥11 and <15 years (n=459)	≥15 and <20 years (n=663)		
IL-10	1.18±0.17	1.22±0.16	-3.851	**<0.001**	17.22±6.81	16.65±6.42	1.567	0.234
IL-1β	-0.05±0.23	0.00±0.20	-4.649	**<0.001**	0.94±0.40	1.11±0.47	-5.148	**<0.001**
IL-6	0.55±0.33	0.56±0.28	-0.785	0.433	4.32±4.55	5.13±5.37	-2.945	**<0.001**
IL-8	1.28±0.39	1.22±0.40	2.595	**<0.001**	24.00±19.79	26.42±21.80	-1.253	0.234
TNF-α	0.69±0.18	0.62±0.20	6.618	**<0.001**	4.69±1.94	5.21±2.25	-3.463	**<0.001**

Note: * FDR adjusted *P* value; All statistically significant values were bolded.

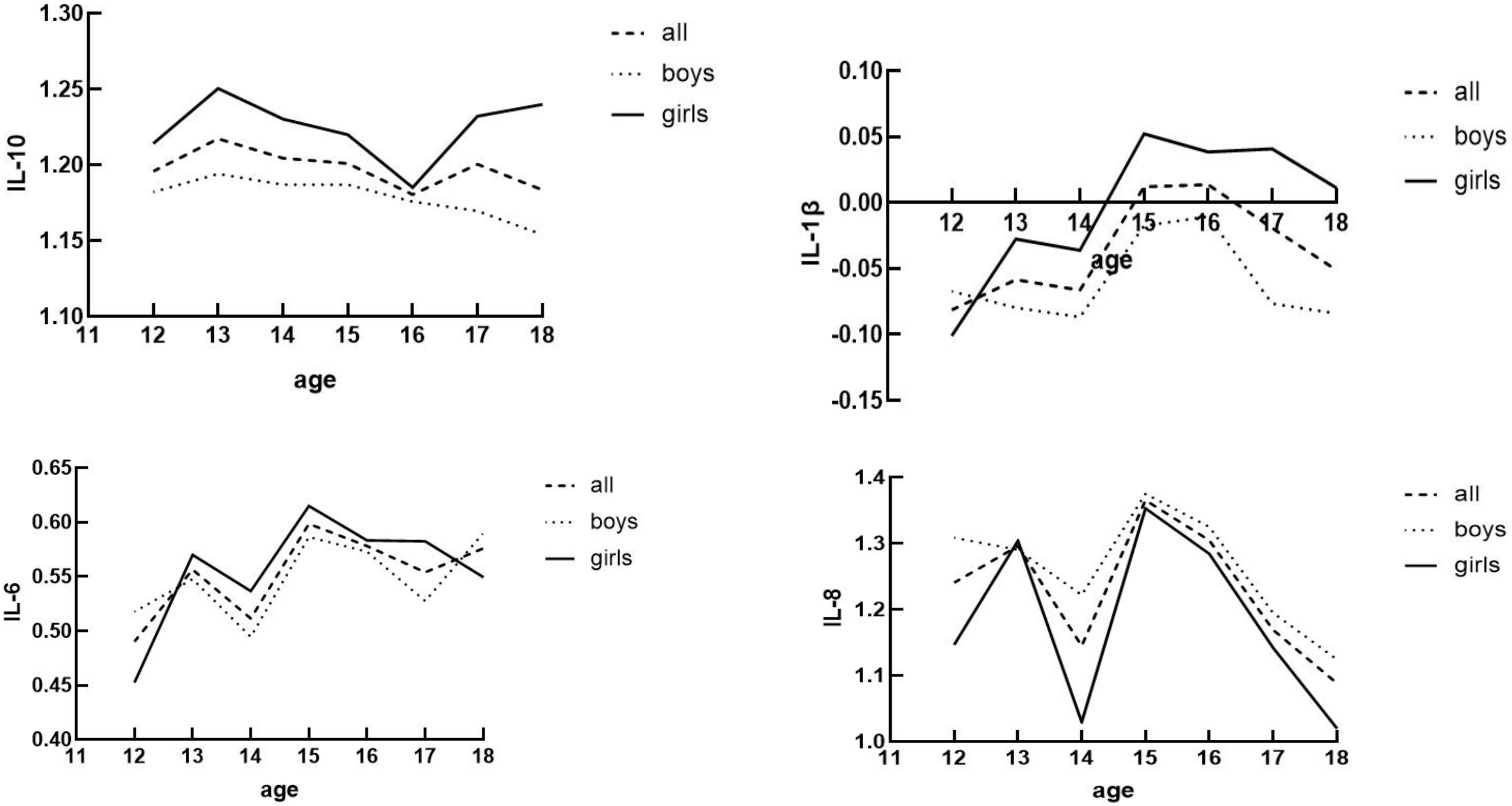
IL-10
1.30
1.25
1.20
1.15
1.10
11
12
13
14
15
16
17
18
age
all
boys
girls
IL-1β
0.10
0.05
0.00
-0.05
-0.10
-0.15
12
13
14
15
16
17
18
age
all
boys
girls
IL-6
0.65
0.60
0.55
0.50
0.45
0.40
11
12
13
14
15
16
17
18
age
all
boys
girls
IL-8
1.4
1.3
1.2
1.1
1.0
11
12
13
14
15
16
17
18
age
all
boys
girls

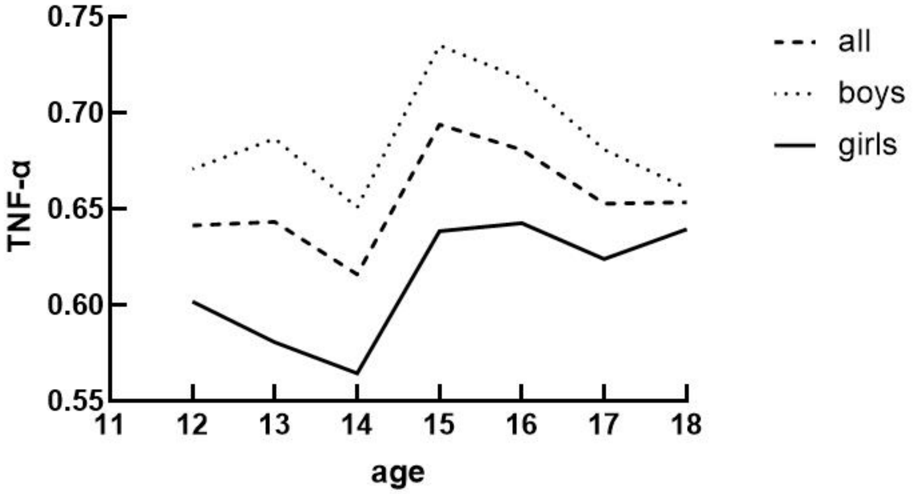

Fig 1 Cytokine distributions by gender and age

Table A2 Associations of childhood maltreatment with the levels of peripheral serum inflammatory cytokines in the total sample

Childhood maltreatment	IL-10					IL-1β					IL-6					IL-8					TNF-α				
	B	95%*CI*	*SE*	*t*	*P**	*B*	95%*CI*	*SE*	*t*	*P**	*B*	95%*CI*	*SE*	*t*	*P**	*B*	95%*CI*	*SE*	*t*	*P**	*B*	95%*CI*	*SE*	*t*	*P**
Emotional abuse	-0.01	-0.08~0.06	0.04	-0.23	1.00	-0.07	-0.16~0.02	0.05	-1.56	0.72	0.15	0.01~.28	0.07	2.13	0.18	0.10	-0.08~0.27	0.09	1.10	0.18	-0.02	-0.10~0.07	0.04	-0.38	1.00
Physical abuse	-0.06	-0.16~0.04	0.05	-1.18	1.00	-0.09	-0.22~0.03	0.07	-1.44	0.75	0.09	-0.10~0.28	0.10	0.93	1.00	0.03	-0.21~0.27	0.12	0.23	1.00	-0.08	-0.19~0.32	0.06	-1.41	0.96
Sexual abuse	-0.04	-0.18~0.11	0.07	-0.48	1.00	-0.03	-0.22~0.16	0.10	-0.31	1.00	0.07	-0.22~0.35	0.15	0.45	1.00	0.04	-0.31~0.38	0.18	0.21	1.00	-0.01	-0.18~0.15	0.08	-0.15	1.00
Emotional neglect	0.03	-0.03~0.08	0.03	0.98	1.00	0.04	-0.03~0.11	0.03	1.15	1.00	0.03	-0.07~0.13	0.05	0.64	1.00	0.09	-0.04~0.21	0.06	1.36	1.00	0.01	-0.05~0.07	0.03	0.27	1.00
Physical neglect	-0.03	-0.10~0.04	0.03	-0.93	1.00	0.02	-0.07~0.11	0.04	0.46	1.00	0.01	-0.11~0.14	0.06	0.20	1.00	-0.00	-0.16~0.16	0.08	-0.04	1.00	-0.01	-0.09~0.06	0.04	-0.35	1.00
Childhood maltreatment	-0.01	-0.11~0.08	0.05	-0.25	1.00	-0.01	-0.14~0.11	0.06	-0.20	1.00	0.09	-0.09~0.27	0.09	0.96	1.00	0.12	-0.11~0.35	0.12	1.06	1.00	-0.02	-0.13~0.09	0.06	-0.40	1.00

Note: * FDR adjusted *P* value; All statistically significant values were bolded.

Table A3 Gender-specific associations of childhood maltreatment with the levels of peripheral serum inflammatory cytokines

Childhood maltreatment	IL-10					IL-1β					IL-6					IL-8					TNF-α				
	B	95%*CI*	*SE*	*t*	*P**	*B*	95%*CI*	*SE*	*t*	*P**	*B*	95%*CI*	*SE*	*t*	*P**	*B*	95%*CI*	*SE*	*t*	*P**	*B*	95%*CI*	*SE*	*t*	*P**
**Boys**																									
Emotional abuse	0.02	-0.08~0.12	0.05	0.37	1.00	-0.01	-0.14~0.12	0.07	-0.09	1.00	0.20	0.00~0.39	0.10	2.00	0.28	0.08	-0.15~0.32	0.12	0.71	1.00	-0.07	-0.17~0.04	0.05	-1.19	1.00
Physical abuse	-0.06	-0.18~0.06	0.06	-0.96	1.00	-0.09	-0.25~0.07	0.08	-1.11	1.00	0.04	-0.20~0.28	0.12	0.32	1.00	-0.09	-0.37~0.19	0.14	-0.63	1.00	-0.08	-0.21~0.05	0.07	-1.18	1.00
Sexual abuse	-0.04	-0.19~0.12	0.08	-0.45	1.00	-0.03	-0.24~0.18	0.11	-0.29	1.00	0.07	-0.25~0.40	0.17	0.45	1.00	-0.02	-0.39~0.35	0.19	-0.09	1.00	-0.04	-0.22~0.13	0.09	-0.50	1.00
Emotional neglect	-0.01	-0.08~0.06	0.04	-0.16	1.00	0.05	-0.05~0.14	0.05	0.97	1.00	0.00	-0.14~0.14	0.07	0.03	1.00	0.07	-0.09~0.24	0.08	0.87	1.00	-0.00	-0.08~0.07	0.04	-0.08	1.00
Physical neglect	-0.08	-0.17~0.01	0.04	-1.80	0.42	0.03	-0.08~0.15	0.06	0.57	1.00	-0.01	-0.19~0.16	0.09	-0.15	1.00	0.02	-0.19~0.23	0.11	0.20	1.00	0.00	-0.09~0.10	0.05	0.03	1.00
Childhood maltreatment	-0.06	-0.19~0.07	0.06	-0.93	1.00	0.01	-0.16~0.18	0.09	0.14	1.00	0.04	-0.21~0.29	0.13	0.34	1.00	0.09	-0.21~0.38	0.15	0.57	1.00	-0.04	-0.18~0.09	0.07	-0.62	1.00
**Girls**																									
Emotional abuse	-0.03	-0.13~0.07	0.05	-0.57	1.00	**-0.16**	**-0.28~-0.03**	**0.06**	**-2.48**	**0.06**	0.08	-0.10~0.26	0.09	0.84	1.00	0.08	-0.17~0.34	0.13	0.63	1.00	0.04	-0.09~0.17	0.06	0.63	1.00
Physical abuse	-0.06	-0.24~0.12	0.09	-0.64	1.00	-0.20	-0.43~0.03	0.12	-1.69	0.45	0.18	-0.16~0.52	0.17	1.03	1.00	0.29	-0.19~0.76	0.24	1.20	1.00	-0.11	-0.34~0.12	0.12	-0.94	1.00

Sexual abuse	0.00	-0.40~0.41	0.21	0.01	1.00	-0.11	-0.63~0.40	0.26	-0.44	1.00	-0.02	-0.80~0.77	0.40	-0.04	1.00	0.33	-0.71~1.37	0.53	0.62	1.00	0.19	-0.32~0.70	0.26	0.74	1.00
Emotional neglect	0.07	-0.01~0.14	0.04	1.78	0.48	0.04	-0.06~0.13	0.05	0.80	1.00	0.08	-0.06~0.22	0.07	1.16	1.00	0.11	-0.08~0.30	0.10	1.17	1.00	0.02	-0.07~0.12	0.05	0.49	1.00
Physical neglect	0.05	-0.05~0.15	0.05	0.93	1.00	0.00	-0.12~0.13	0.07	0.05	1.00	0.07	-0.11~0.26	0.09	0.77	1.00	-0.03	-0.29~0.23	0.13	-0.22	1.00	-0.04	-0.17~0.08	0.07	-0.67	1.00
Childhood maltreatment	0.07	-0.07~0.21	0.07	0.94	1.00	-0.05	-0.23~0.13	0.09	-0.59	1.00	0.16	-0.10~0.43	0.13	1.21	1.00	0.17	-0.20~0.54	0.19	0.92	1.00	0.01	-0.18~0.19	0.09	0.05	1.00

Note: * FDR adjusted *P* value; All statistically significant values were bolded.

Table A4 Age-specific associations of childhood maltreatment with the levels of peripheral serum inflammatory cytokines

Childhood maltreatment	IL-10					IL-1β					IL-6					IL-8					TNF-α				
	B	95%*CI*	*SE*	*t*	*P**	*B*	95%*CI*	*SE*	*t*	*P**	*B*	95%*CI*	*SE*	*t*	*P**	*B*	95%*CI*	*SE*	*t*	*P**	*B*	95%*CI*	*SE*	*t*	*P**
**⩾11 and <15 years**																									
Emotional abuse	-0.01	-0.11~0.10	0.05	-0.11	1.00	-0.14	-0.26~-0.01	0.07	-2.11	0.24	0.09	-0.10~0.27	0.09	0.93	1.00	-0.18	-0.42~0.06	0.12	-1.51	0.78	-0.01	-0.12~0.10	0.06	-0.17	1.00
Physical abuse	-0.10	-0.22~0.03	0.06	-1.54	0.72	-0.08	-0.23~0.08	0.08	-0.97	1.00	-0.04	-0.25~0.18	0.11	-0.33	1.00	-0.09	-0.38~0.21	0.15	-0.58	1.00	-0.06	-0.19~0.08	0.07	-0.84	1.00
Sexual abuse	-0.02	-0.21~0.18	0.10	-0.17	1.00	-0.13	-0.38~0.12	0.13	-1.00	1.00	0.02	-0.34~0.37	0.18	0.09	1.00	-0.13	-0.60~0.34	0.24	-0.55	1.00	-0.08	-0.30~0.14	0.11	-0.74	1.00
Emotional neglect	0.03	-0.05~0.10	0.04	0.67	1.00	0.04	-0.06~0.14	0.05	0.80	1.00	0.02	-0.12~0.15	0.07	0.25	1.00	-0.04	-0.22~0.14	0.09	-0.43	1.00	0.06	-0.02~0.14	0.04	1.39	1.00
Physical neglect	-0.04	-0.14~0.06	0.05	-0.76	1.00	-0.00	-0.13~0.13	0.06	-0.02	1.00	0.02	-0.15~0.20	0.09	0.26	1.00	-0.12	-0.36~0.11	0.12	-1.01	1.00	-0.03	-0.13~0.08	0.06	-0.46	1.00
Childhood maltreatment	-0.03	-0.17~0.10	0.07	-0.46	1.00	-0.08	-0.25~0.10	0.09	-0.86	1.00	0.02	-0.23~0.26	0.12	0.12	1.00	-0.21	-0.53~0.11	0.16	-1.29	1.00	0.02	-0.13~0.17	0.08	0.25	1.00
**⩾15 and＜20 years**																									
Emotional abuse	0.00	-0.09~0.10	0.05	0.03	1.00	-0.03	-0.15~0.10	0.06	-0.41	1.00	0.19	0.00~0.38	0.10	2.00	0.23	**0.39**	**0.16~0.63**	**0.12**	**3.26**	**0.01**	-0.01	-0.12~0.11	0.06	-0.12	1.00
Physical abuse	0.01	-0.15~0.17	0.08	0.08	1.00	-0.11	-0.32~0.10	0.11	-1.02	1.00	0.37	0.04~0.69	0.17	2.18	0.18	0.35	-0.04~0.74	0.20	1.78	0.24	-0.08	-0.26~0.11	0.10	-0.78	1.00
Sexual abuse	-0.05	-0.25~0.16	0.11	-0.43	1.00	0.06	-0.21~0.33	0.14	0.43	1.00	0.13	-0.32~0.58	0.23	0.56	1.00	0.24	-0.27~0.74	0.26	0.92	0.58	0.06	-0.19~0.30	0.13	0.47	1.00
Emotional neglect	0.03	-0.04~0.10	0.04	0.83	1.00	0.04	-0.05~0.13	0.05	0.81	1.00	0.04	-0.09~0.18	0.07	0.60	1.00	**0.20**	**0.04~0.37**	**0.09**	**2.37**	**0.08**	-0.03	-0.11~0.06	0.04	-0.62	1.00
Physical neglect	-0.02	-0.11~0.07	0.05	-0.37	1.00	0.02	-0.09~0.14	0.06	0.38	1.00	0.01	-0.17~0.19	0.09	0.08	1.00	-0.04	-0.10~0.03	0.11	1.07	0.58	-0.01	-0.12~0.10	0.05	-0.16	1.00
Childhood maltreatment	0.02	-0.11~0.15	0.07	0.30	1.00	0.03	-0.14~0.20	0.09	0.32	1.00	0.16	-0.10~0.43	0.13	1.23	0.88	**0.50**	**0.18~0.82**	**0.16**	**3.06**	**0.01**	-0.05	-0.20~0.11	0.08	-0.57	1.00

Note: * FDR adjusted *P* value; All statistically significant values were bolded.
